# Bionic reconstruction of tension trabeculae in short-stem hip arthroplasty: a finite element analysis

**DOI:** 10.1186/s12891-023-06205-3

**Published:** 2023-02-02

**Authors:** Zhentao Ding, Jun Wang, Yanhua Wang, Xiaomeng Zhang, Yong Huan, Dianying Zhang

**Affiliations:** 1 Department of Orthopedics and Trauma, Peking University People’s Hospital, Beijing, 100044 China; 2 National Centre for Trauma Medicine, Peking University People’s Hospital, Beijing, 100044 China; 3 Key Laboratory of Trauma and Neural Regeneration (Peking University), Ministry of Education, Beijing, 100044 China; 4 State Key Laboratory of Nonlinear Mechanics (LNM), Institute of Mechanics, Chinese Academy of Sciences, Beijing, 100190 China; 5 School of Engineering Science, University of Chinese Academy of Sciences, Beijing, 100049 China

**Keywords:** Short-stem hip arthroplasty, Stress shielding, Hip biomechanics, Bionic reconstruction, Tension screw, Finite element analysis

## Abstract

**Background:**

Short-stem hip arthroplasty (SHA) is characterized by metaphyseal load transfer that effectively preserves the bone stock, but still suffers from stress shielding in the proximal femur. We designed a tension screw to mimic tension trabeculae in the new bionic collum femoris preserving (BCFP) short stem for bionic reconstruction, aiming to restore the biomechanics of hip joint.

**Methods:**

Native femur finite element model was constructed to investigate the biomechanics of hip joint based on computed tomography (CT) data. The maximum absolute principal stress/strain cloud chart allowed the direction of stress/strain to be assessed. Six BCFP models with different screw angles (5°, 10°, 15°, 20°, 25°, and 30°) and the Corail model were created. The stress/strain distribution and overall stiffness were compared between each of the BCFP and Corail implanted models.

**Results:**

The native model visualized the transfer pathways of tensile and compressive stress. The BCFP stems showed significantly higher stress and strain distribution in the greater trochanteric region compared to conventional total hip arthroplasty (THA). In particular, the BCFP-5° stem demonstrated the highest average strain in both medial and lateral regions and the overall stiffness was closest to the intact femur.

**Conclusions:**

Stress transfer pathways of trabecular architecture provide biomechanical insight that serves as the basis for bionic reconstruction. The tension screw improves load transfer pattern in the proximal femur and prevents stress reduction in the greater trochanteric region. The BCFP-5° stem minimizes the stress shielding effect and presents a more bionic mechanical performance.

## Background

Young and active patients remain challenging for total hip arthroplasty (THA), where periprosthetic osteolysis and aseptic loosening are critical factors affecting the long-term survival of implants. Mismatch between the implant and the proximal femur often produces stress shielding effect, creating a non-physiological stress distribution [1]. Reduced load transfer induces periprosthetic bone loss and is one of the major reasons for cementless implant failure [2].

Standard THA with distal fixation leads to proximal stress shielding [3]. Short-stem hip arthroplasty (SHA) were developed as a bone conserving solution. Its design concept is to preserve bone tissue, optimize physiological load transfer and limit stress to the metaphysis based on the anchoring principle [4, 5]. The advantage of SHA over standard THA is that the implant-bone interface only lies in the proximal region, surrounded by cancellous bone, which reduces bone loss due to stress shielding [6]. It is worth noting that although short- and mid-term follow-up studies have indicated favorable outcomes, SHA does not offer higher survivorship than conventional THA [7, 8]. Dual energy X-ray absorptiometry (DEXA) is a common method for measuring bone mineral density (BMD) and periprosthetic area division is widely applied to evaluate proximal femoral implants [9]. Researchers also discovered that bone remodeling occurred primarily in the metaphysis and distal region by DEXA, while stress shielding in the proximal region was unavoidable [10, 11]. These facts suggest that the existing short-stemmed implants still need further optimization.

Previous studies described the stress-dispersing role of proximal femoral trabecular architecture from a morphological and biomechanical perspective [12, 13]. Existing short stems only focus on implant-metaphysis fitting, relying on the femoral calcar and distal lateral cortex for fixation and load transfer, whereas bone atrophy occurs in the greater trochanteric region [14, 15]. This leads to the assumption that short stems ignore the stress-dispersing effect of tension trabeculae, with consequent unloading of the proximal region, particularly the greater trochanter [16].

The purpose of this study was to investigate whether the bionic reconstruction of tension trabeculae with a tension screw could reduce the stress shielding effect in SHA. We hypothesized that the bionic reconstruction would contribute to restore the biomechanics of hip joint. Prior to this, we investigated the biomechanics of trabecular architecture, by visualizing the compression and tension zones through finite element (FE) analysis.

## Materials and methods

### Implant design

**Fig. 1:**
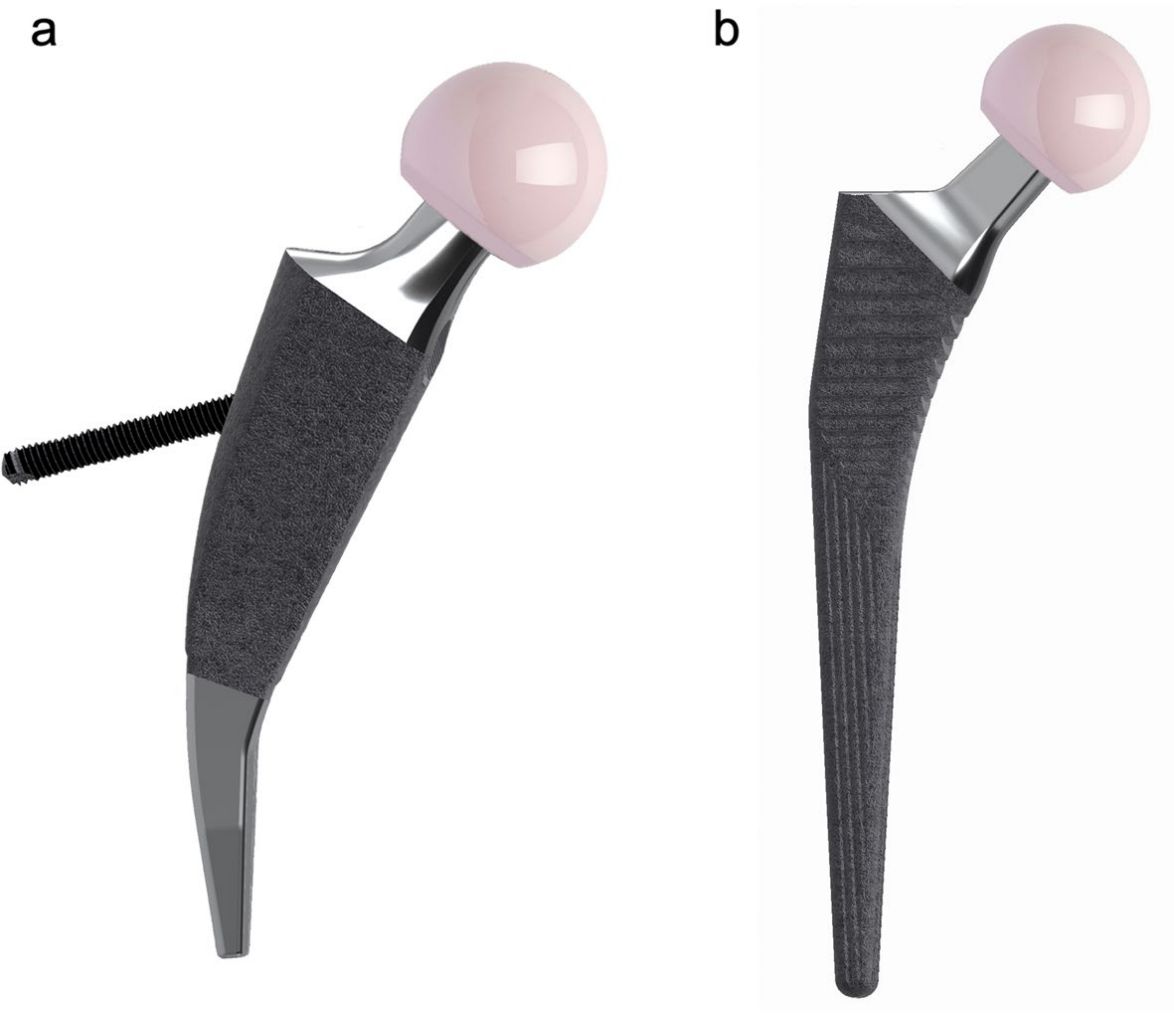
Implant design. **a** BCFP stem, a cementless calcar-loading short-stemmed implant with a tension screw. **b** Corail stem, a cementless standard straight implant

The Metha® short stem (B. Braun, Aesculap, Tuttlingen, Germany) is a cementless calcar-loading implant with bone ingrowth coating on the proximal and middle portions [17]. According to the stress dispersion hypothesis of tension trabeculae, the new short stem was designed with a tension screw based on Metha®. The head end of tension screw is positioned medial to the neck-body junction of stem, with the screw passing through the stem body and the tail end fixed to the lateral femoral cortex (Fig. 1a). Under loading conditions, the femoral stem tends to be compressed and the tension screw tends to be tensioned, resulting in a more physiological stress distribution in the proximal femur. When the tension screw is perpendicular to the distal axis of stem, the screw angle is defined as 0°. It is conceived both for the feasibility of intraoperative manipulation and screw insertion. Therefore, the screw angles were set to 5°, 10°, 15°, 20°, 25°, and 30°, and the screw diameter was set to 6 mm to create six implant models. The tension screw mimics tension trabeculae to provide a bionic reconstruction of the proximal femur and the osteotomy partially preserves the femoral neck, so that the new short-stemmed implant is named bionic collum femoris preserving (BCFP).

The Corail® standard stem (DePuy International Ltd, Leeds, UK) is a cementless straight implant covered with a full hydroxyapatite coating (Fig. 1b). It has been in clinical use for over 30 years with excellent long-term results [18]. According to the volunteer femoral model, both prosthetic stems were selected as size 3 among sizes 0–6, with a 135° neck-shaft angle.

### FE models

**Fig. 2:**
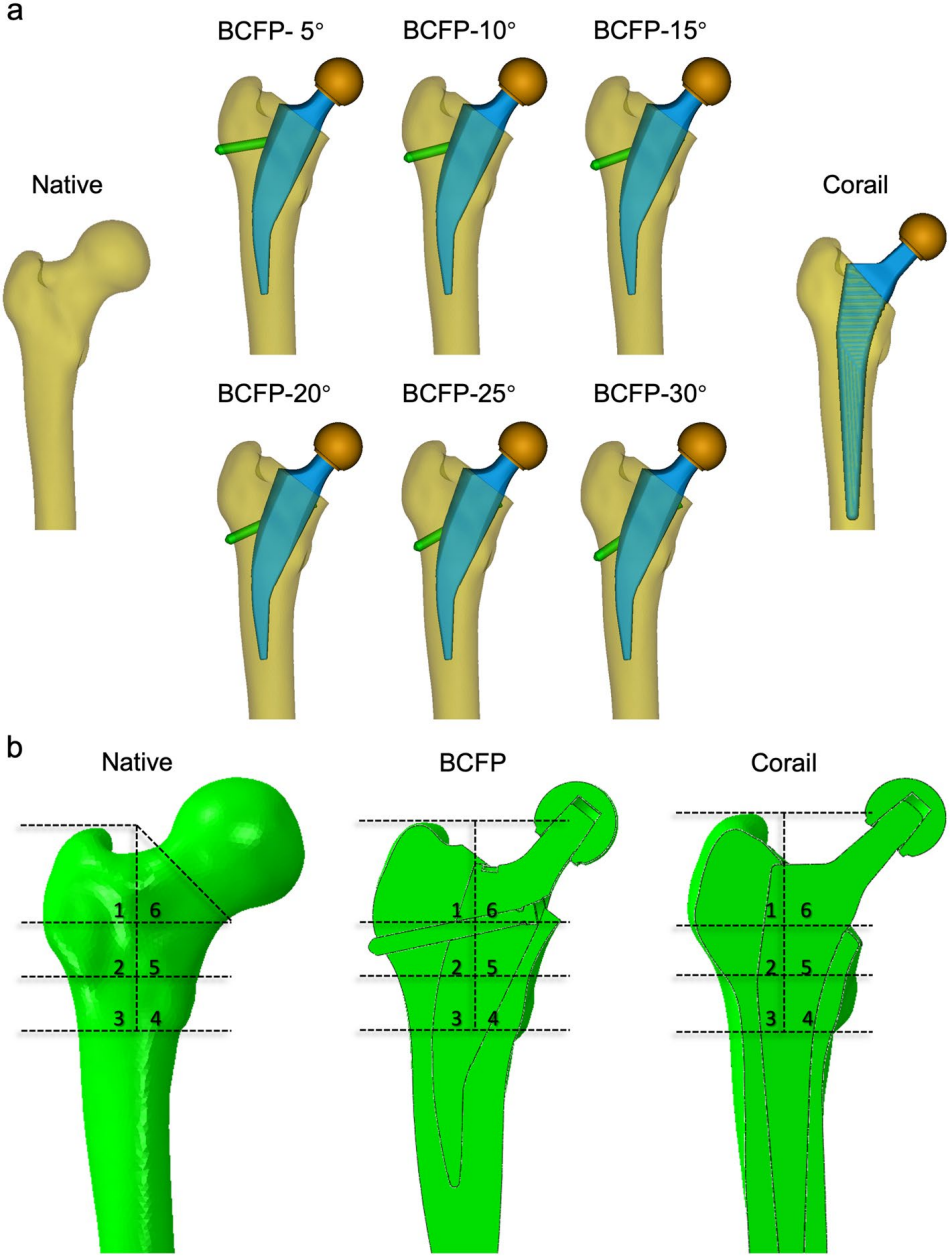
**a** Native model and implanted models with BCFP and Corail. **b** Region division, each model was divided into six ROIs from the tip of greater trochanter to the base of lesser trochanter

The native femur model was based on computed tomography (CT) data of the right femur of a 58-year-old male volunteer with no history of severe trauma. The images were acquired by a 64-slice CT scanner (LightSpeed VCT, GE Healthcare, Milwaukee, WI, USA) with the slice thickness of 1.0 mm. The data in DICOM format were imported into Mimics 19.0 (Materialise, Leuven, Belgium) and the three-dimensional femur model was constructed by automatic threshold-based segmentation. The osteotomy level was partial collum for the BCFP stem and trochanter sparing for the Corail stem [19]. Following the standard surgical procedure, the femur models were virtually osteotomized and the prosthetic stems were respectively implanted (Fig. 2a). The center of prosthetic head was located at the center of femoral head and the axis of prosthetic stem was parallel to the axis of femoral shaft. The FE models were meshed by Hypermesh 12.0 (Altair Engineering GmbH, Böblingen, Germany). The element type was a 4-node linear tetrahedron (C3D4) and the average element size was 1.35 mm according to mesh convergence analysis.

### Material properties

Pre-processing and solving were performed using Abaqus 16.0 (Simulia, Dassault Systèmes, Vélizy-Villacoublay, France). The femur model assignment was divided into 10 gradients by CT Hounsfield unit (HU) values, demonstrating the inhomogeneous material properties of bone. For non-flexible stems, different density-modulus relationships have no significant effect on the stress shielding effect [20]. The HU values for each gradient were therefore converted to elastic modulus with the following linear relationship [21, 22]:

1 $$E=8.28\times CT+92$$

where *E* is the elastic modulus (MPa) and *CT* represents the HU value. The cementless implants and tension screws are forged from titanium alloy. The implant material was assumed to be homogeneous and isotropic and its mechanical behavior was simulated with a linear-elastic model. The elastic modulus of the implant material was set at 110 G. The Poisson’s ratio was set at 0.34 for both the femur and the implant material [23].

### Boundary and loading conditions

The cementless implants are subject to reversible micromotion and irreversible migration at the initial stage. As bone ingrowth is promoted, the initial stability obtained by press-fitting transitions to secondary stability. Thereafter, the micromotion and migration gradually settle down [24, 25]. In order to simulate the long-term situation after bone ingrowth, the implant-bone interface was set up in this study as a tied contact with no relative micromotion. The distal femur was constrained in all directions.

The loading configuration simulated a single-legged stance. A weight-related load of 347% of body weight was applied to the same reference point at the prosthetic neck in the BCFP and Corail implanted models [26]. The same load was applied to the top of femoral head in the native model. In the present study, the volunteer weighed 75 kg and the load was 2600 N. The loading vector was configured as a mechanical alignment of the lower limb, pointing from the hip joint center to the knee joint center [27]. This implies that in addition to the load along the anatomical alignment of femur, there are also certain anterior and lateral load components.

### Statistical analysis

A nonlinear static analysis of the FE models was performed. The native model was used to investigate the biomechanics of hip joint. The maximum absolute principal stress/strain cloud chart allowed the direction of stress/strain to be assessed. The von Mises stress and strain distribution were compared between each of the BCFP and Corail implanted models. To quantify the stress and strain distribution at the proximal femur, six regions of interest (ROI) were divided from the tip of greater trochanter to the base of lesser trochanter (Fig. 2b). The proximal (region 1) and surrounding (region 2) regions of tension screw correspond to the greater trochanteric region. The stress and strain data were extracted from each element and the average value of each ROI was calculated. In addition, force–displacement curves were plotted for each model to examine the variations in overall stiffness caused by the implantation of different stems.

Statistical analysis was carried out using GraphPad Prism 9 (GraphPad Software, San Diego, USA). The Kolmogorov–Smirnov test showed that the stress and strain data for each ROI did not conform to the normal distribution. These continuous variables were expressed as the median and interquartile range. To further compare the data distribution between groups, a non-parametric one-way ANOVA was performed using the Kruskal–Wallis test and Dunn's multiple comparison test. The significance level was set at 0.05.

## Results

### Native model

**Fig. 3:**
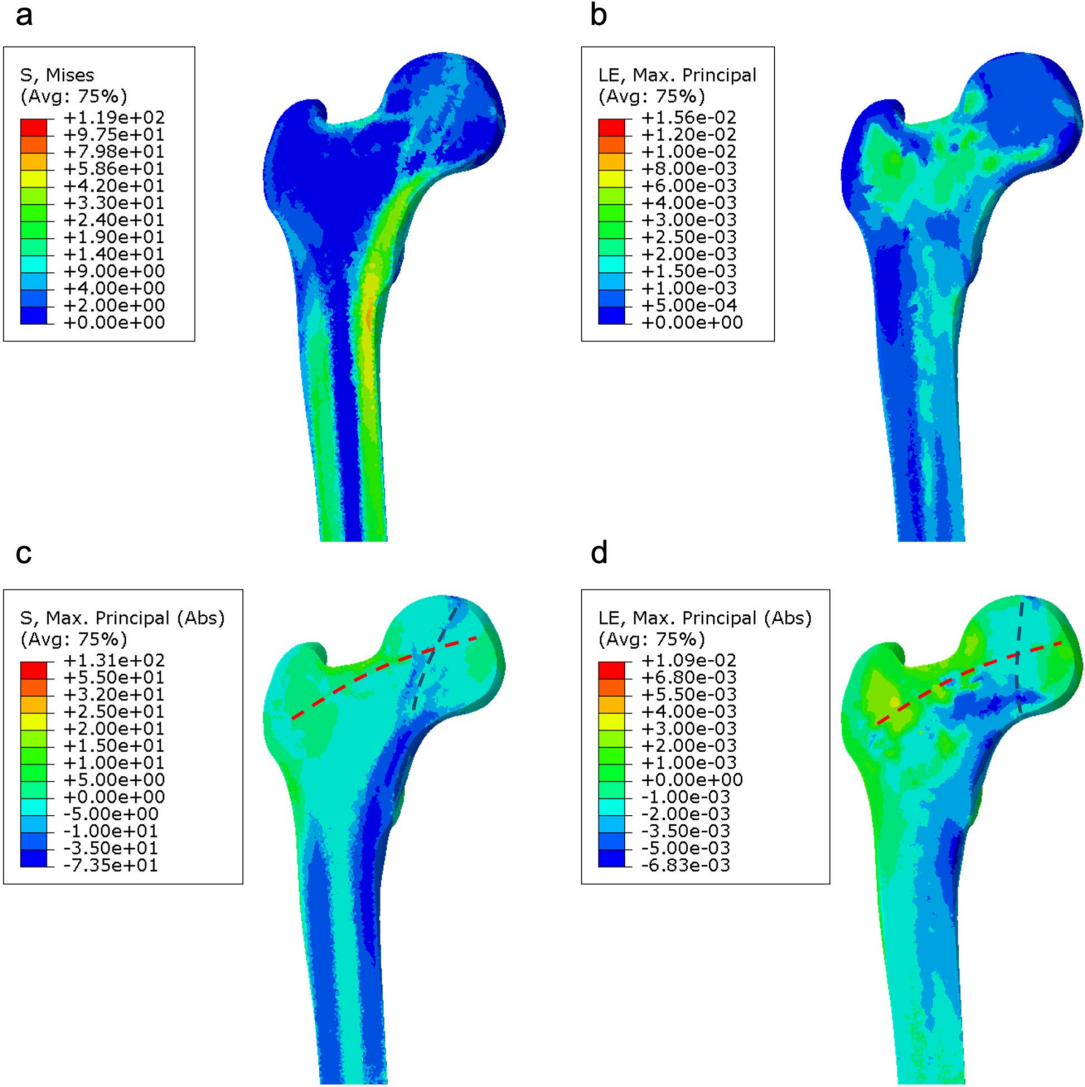
Native femur FE model. **a** Von Mises stress cloud chart, **b** strain cloud chart, **c** maximum absolute principal stress cloud chart (positive values for tensile stress and negative values for compressive stress), and (**d**) maximum absolute principal strain cloud chart (positive values for tensile deformation and negative values for compressive deformation). The red dashed line represents tensile stress transfer pathway and the blue dashed line represents compressive stress transfer pathway

In von Mises stress cloud chart, a distinct stress zone within the femoral head could be observed in the native model, oriented approximately in the direction of mechanical alignment (Fig. 3a). The strain values in the femoral neck and greater trochanteric region were higher than other regions, around 3000 µm/m (Fig. 3b). The native model demonstrated the tensile (red dashed line) and the compressive (blue dashed line) stress transfer pathway in maximum absolute principal stress/strain cloud chart (Fig. 3c and d). The tensile stress was transmitted from the medial femoral head through the femoral neck to the greater trochanter. The compressive stress was transmitted from the superior femoral head through the medial trabecular column to the calcar.

### Stress distribution

**Fig. 4:**
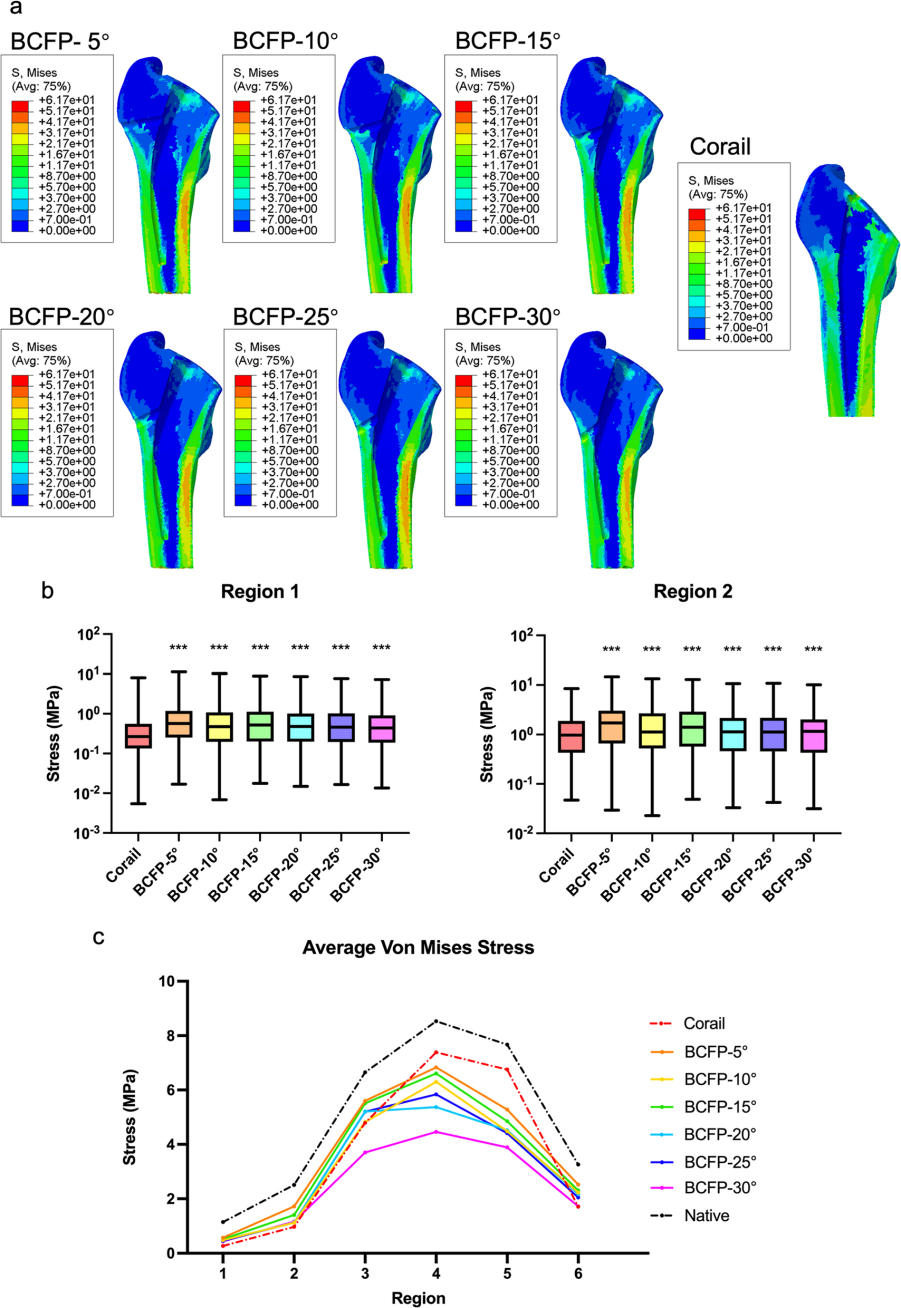
**a** Von Mises stress cloud chart for implanted models. **b** Comparison of von Mises stress distribution in the greater trochanter (region 1–2) between the BCFP and Corail models. **c** Comparison of the average von Mises stress in six ROIs for the native and implanted models. *p < 0.05, **p < 0.01, ***p < 0.001, non-parametric one-way ANOVA

The BCFP and Corail implanted models presented different stress distribution patterns to the native femur, with clearly reduced stress following implantation (Fig. 4a). The stress distribution around and inferior to tension screw was increased in the BCFP models. As the screw angle decreased, the area of increased stress in the greater trochanter became larger.

In the greater trochanter (region 1–2), the von Mises stress distribution was significantly higher for all BCFP models with different screw angles than the Corail model (Fig. 4b; *p* < 0.001). Comparing the average von Mises stress across six ROIs, the Corail model concentrated stress transfer mainly on the medial femur (region 4–5) (Fig. 4c). The BCFP models partially transferred stress from the medial to the lateral femur (region 1–3). The BCFP-5° model had the highest average stress in region 1–3. The BCFP-30° model presented a lower level of average stress in all ROIs.

### Strain distribution

**Fig. 5:**
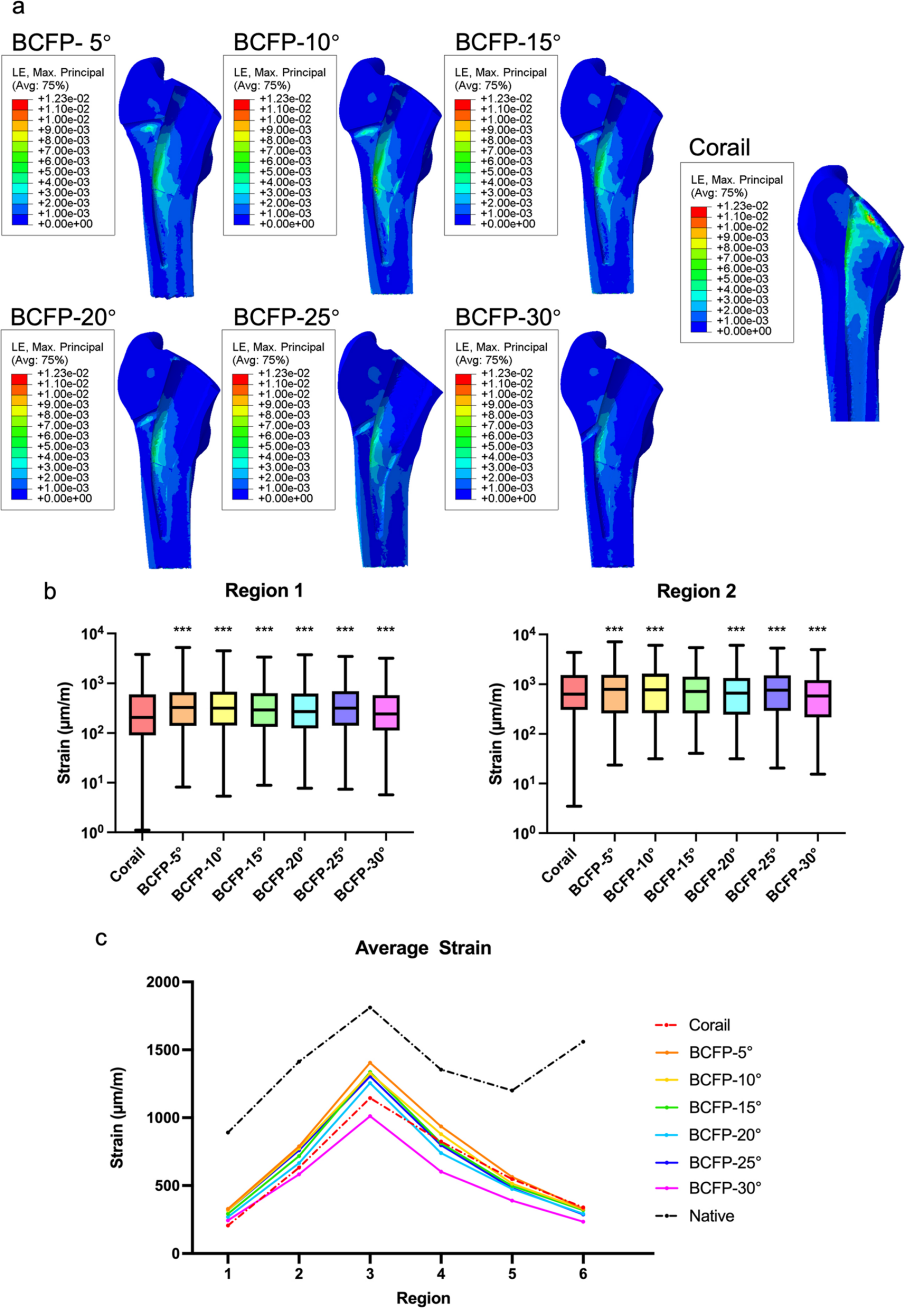
**a** Strain cloud chart for implanted models. **b** Comparison of strain distribution in the greater trochanter (region 1–2) between the BCFP and Corail models. **c** Comparison of the average strain in six ROIs for the native and implanted models.*p < 0.05, **p < 0.01, ***p < 0.001, non-parametric one-way ANOVA

At the macroscopic scale, there was no visual difference between the strain and stress distribution of implanted models (Fig. 5a). The strain distribution also increased at the implant-bone interface around and inferior to tension screw in the BCFP models. The screw angle determined the extent of strain increasing area in the greater trochanter.

In region 1, the strain distribution for all BCFP models was significantly higher than the Corail model (Fig. 5b; *p* < 0.001). In region 2, the BCFP-5°, BCFP-10°, BCFP-20°, and BCFP-25° models demonstrated higher strain distribution compared to standard THA (Fig. 5b; *p* < 0.001). Among six ROIs, the Corail model displayed moderate average strain in the medial femur (region 4–5) (Fig. 5c). In the lateral femur (region 1–3), the average strain in all BCFP models except BCFP-30° exceeded the Corail model. The BCFP-5° model had the highest average strain in region 1–5. The BCFP-30° model also presented a lower level of average strain in all ROIs.

### Stiffness

**Fig. 6:**
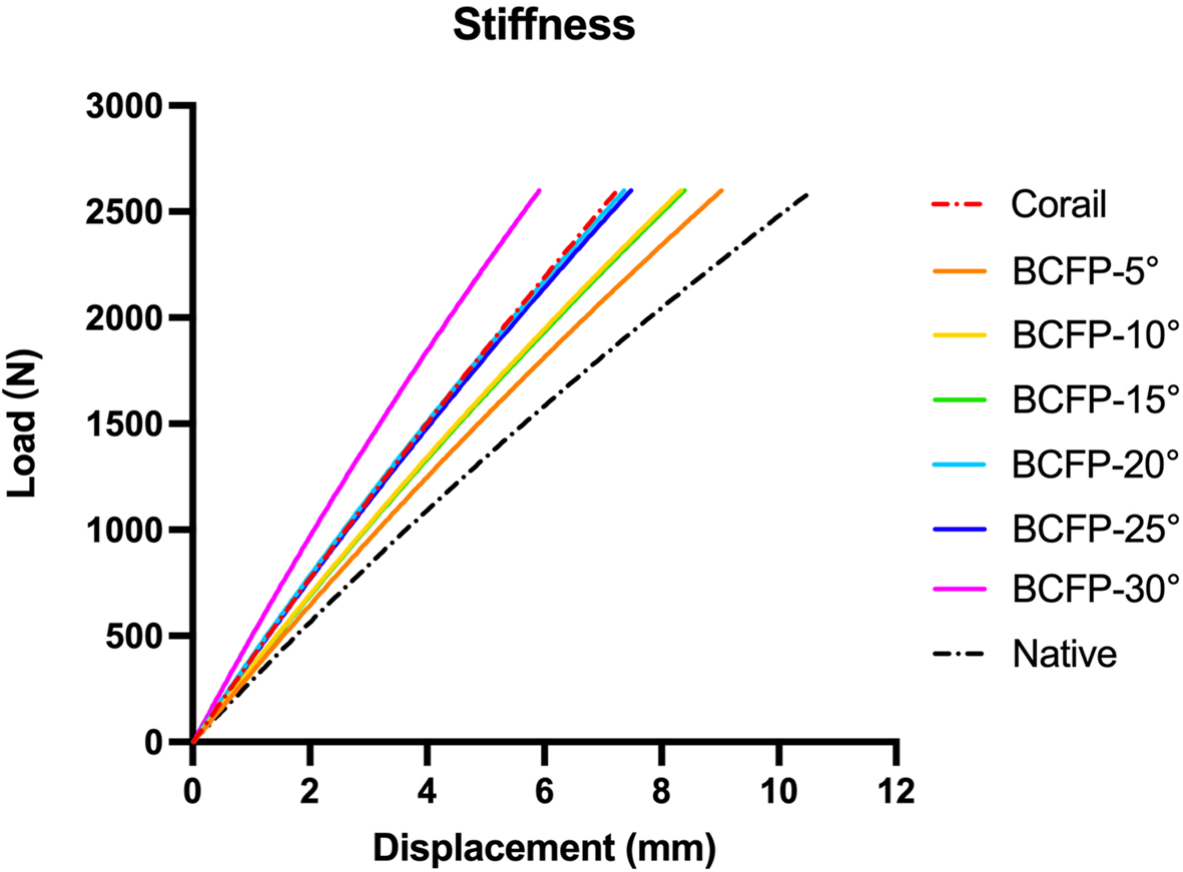
Comparison of force–displacement curves for the native and implanted models

The force–displacement curves of implanted models were compared with the native model under the same conditions (Fig. 6). The curve of BCFP-5° model was the closest to intact femur, with the least variation in overall stiffness due to implantation. The overall stiffness of BCFP-20°, BCFP-25°, and Corail models were similar and at a moderate level, while the BCFP-30° implant induced the most variation.

## Discussion

In this study, the bionic reconstruction of tension trabeculae was achieved with tension screw, which enhanced the load transfer pattern of the proximal femur and improved the stress/strain distribution in the greater trochanteric region. In particular, the BCFP-5° implant showed the highest average strain in both medial and lateral femur, with an overall stiffness closest to the native femur, and performed optimally in stress shielding prevention.

Wolff depicted the morphological architecture of tension and compression trabeculae by observing the human femur [13]. In computational simulation, the adaptive remodeling of trabecular architecture was consistent with the actual femur when multiple-loading conditions were applied similar to the reality [12]. The stress transfer pathways demonstrated in this study by the native FE model are similar to previous morphological and biomechanical studies, thereby validating the biomechanical properties of tension and compression trabeculae.

A finite element study by Ong et al. indicated that the stress in native femur was transferred from the superior femoral head through the center of head to the medial column [28]. This is in accordance with the compressive stress transfer pathway in the present study. Multiple investigations analyzed the load transfer pattern based only on the magnitude of von Mises stress, regardless of the stress direction [29, 30]. As a result, the stress dispersion effect of tension trabeculae is prone to be neglected [31]. In our analysis, tensile stress transfer pathway was complemented by the distinction of stress direction through maximum absolute principal stress/strain cloud chart. This insight into the biomechanics of hip joint provides the basis for bionic reconstruction concept in cementless stem design.

Stress shielding and bone remodeling are important indicators for evaluating implant design, both depending on the fixed position of stem [32]. Conventional THA is characterized by distal anchoring and proximal unloading and the short stem design is based on the metaphyseal anchoring concept [33, 34]. Prior finite element studies proved that SHA demonstrated a more physiological load transfer in the proximal and metaphysis [35]. Besides, DEXA investigations found more balanced BMD changes and less BMD loss after SHA [10, 36]. However, the existing short stems are still unable to completely avoid stress reduction in the greater trochanter [37, 38]. To further optimization, the new short stem should have a more bionic design. In the BCFP stem, tension screw is used to mimic tension trabeculae for bionic reconstruction. The results suggested that this design improved the load transfer in the greater trochanteric region. This is probably due to the lever effect of tension screw, which partially transmits the stress from the medial femur to the lateral, providing a bionic stress distribution.

In the BCFP stem, the tension screw angle is also a key factor impacting on the biomechanical properties. As shown in Figs. 4 and 5, the BCFP-5° stem performed best in terms of reducing the stress shielding effect. In contrast, the BCFP-30° model had a lower stress/strain distribution in all regions of the proximal femur. This difference may depend on the relative position between the tension screw and the greater trochanter. At a screw angle of 5°, the BCFP stem features a more proximal anchorage and presents a more physiological load transfer. Accordingly, we speculate that the biomechanical properties of tension screw in BCFP-5° stem is closest to the tension trabecular architecture, presenting the implant with the most bionic design.

Limitations arose mainly from the simplifications and assumptions of FE models. The simplified loading configurations did not simulate muscle strength and to a certain extent differed from clinical practice [39]. In the present study, we only assessed the conditions after bone ingrowth, not the initial stability of implants, and were unable to simulate the dynamic process of trabecular remodeling. Besides, the screw placement may break the lateral femoral cortex and possibly greatly increase the operating time as well. Furthermore, the thread connection between tension screw and prosthesis in the BCFP stem creates a new interface. Previous reviews reported no significant difference in revision rates between modular and non-modular hip implants [17]. But interfacial corrosion and connection fatigue in modular implants are still a concern for surgeons.

## Conclusions

This finite element study investigated the combined effect of short-stemmed implant and tension screw on the load transfer pattern. The BCFP stem was based on a metaphyseal anchoring short stem and the tension trabeculae were bionically reconstructed by a tension screw. The results showed that the tension screw partially transferred the stress from the medial femur to the lateral, significantly improving the stress/strain distribution in the greater trochanteric region. The BCFP -5° configuration showed best results in terms of stress distribution. Nevertheless, further biomechanical experiments and clinical studies are still required to evaluate bone remodeling of the BCFP stem.

## Data Availability

The datasets and materials are available from corresponding authors on reasonable request.

## Abbreviations

THA: Total hip arthroplasty
SHA: Short-stem hip arthroplasty
BMD: Bone mineral density
DEXA: Dual energy X-ray absorptiometry
FE: Finite element
BCFP: Bionic collum femoris preserving
CT: Computed tomography
HU: Hounsfield unit
ROI: Regions of interest
